# Aquaporin-3a Dysfunction Impairs Osmoadaptation in Post-Activated Marine Fish Spermatozoa

**DOI:** 10.3390/ijms25179604

**Published:** 2024-09-04

**Authors:** François Chauvigné, Júlia Castro-Arnau, Noelia López-Fortún, Alejandro Sánchez-Chardi, Michael Rützler, Giuseppe Calamita, Roderick Nigel Finn, Joan Cerdà

**Affiliations:** 1 Institute of Marine Sciences, Spanish National Research Council (CSIC), 08003 Barcelona, Spain; chauvigne@icm.csic.es (F.C.); julia.c@wustl.edu (J.C.-A.); noelialopez@icm.csic.es (N.L.-F.); nigel.finn@uib.no (R.N.F.); 2 Institute of Biotechnology and Biomedicine (IBB), Universitat Autònoma de Barcelona, Bellaterra, Cerdanyola del Vallès, 08193 Barcelona, Spain; 3 Microscopy Service, Universitat Autònoma de Barcelona, Bellaterra, Cerdanyola del Vallès, 08193 Barcelona, Spain; alejandro.sanchez.chardi@uab.cat; 4 Department of Evolutionary Biology, Ecology and Environmental Sciences, Faculty of Biology, Universitat de Barcelona, 08028 Barcelona, Spain; 5 Apoglyx AB, c/o Anyo AB, Ideon Science Park, 22370 Lund, Sweden; michael.rutzler@apoglyx.com; 6 Department of Biochemistry and Structural Biology, Lund University, 22184 Lund, Sweden; 7 Department of Biosciences, Biotechnologies and Environment, University of Bari “Aldo Moro”, 70125 Bari, Italy; giuseppe.calamita@uniba.it; 8 Department of Biological Sciences, University of Bergen, 5020 Bergen, Norway

**Keywords:** fish, sperm motility, volume regulation, DFP00173

## Abstract

Spermatozoon volume regulation is an essential determinant of male fertility competence in mammals and oviparous fishes. In mammals, aquaporin water channels (AQP3, -7 and -8) have been suggested to play a role in spermatozoon cell volume regulatory responses in the hypotonic female oviduct. In contrast, the ejaculated spermatozoa of marine teleosts, such as the gilthead seabream (*Sparus aurata*), experience a high hypertonic shock in seawater, initially resulting in an Aqp1aa-mediated water efflux, cell shrinkage and the activation of motility. Further regulatory recovery of cell volume in post-activated spermatozoa is mediated by Aqp4a in cooperation with the Trpv4 Ca^2+^ channel and other ion channels and transporters. Using a paralog-specific antibody, here, we show that seabream spermatozoa also express the aquaglyceroporin AQP3 ortholog Aqp3a, which is highly accumulated in the mid posterior region of the spermatozoon flagella, in a similar pattern to that described in mouse and human sperm. To investigate the role of Aqp3a in seabream sperm motility, we used a recently developed AQP3 antagonist (DFP00173), as well as the seabream Aqp3a-specific antibody (α-SaAqp3a), both of which specifically inhibit Aqp3a-mediated water conductance when the channel was heterologously expressed in *Xenopus laevis* oocytes. Inhibition with either DFP00173 or α-SaAqp3a did not affect sperm motility activation but did impair the spermatozoon motion kinetics at 30 s post activation in a dose-dependent manner. Interestingly, in close resemblance to the phenotypes of AQP3-deficient murine sperm, electron microscopy image analysis revealed that both Aqp3a inhibitors induce abnormal sperm tail morphologies, including swelling and angulation of the tail, with complete coiling of the flagella in some cases. These findings suggest a conserved role of Aqp3a as an osmosensor that regulates cell volume in fish spermatozoa under a high hypertonic stress, thereby controlling the efflux of water and/or solutes in the post-activated spermatozoon.

## 1. Introduction

Spermatozoon volume regulation is an essential determinant of male fertility competence in mammals and oviparous fishes [1,2]. In mammals, ejaculated spermatozoon volume initially increases when exposed to the comparatively mild hypotonic conditions (∆^−^ ~100 mOsm) of the female reproductive tract, but subsequently undergoes a regulatory volume decrease (RVD) to maintain the swimming kinematic properties required for further passage into the oviduct [3]. In oviparous freshwater fishes, the hypotonic shock is much greater (∆^−^ ~250 mOsm) and known to be important for motility activation [4,5]; however, mechanisms of RVD remain to be established. By contrast, the ejaculated spermatozoa of oviparous marine fishes experience a strong hypertonic shock in seawater (∆^+^ ~750 mOsm), resulting in an initial shrinkage that activates motility, but they subsequently undergo a regulatory volume increase (RVI) to maintain post-activated swimming performance [6]. In both mammals and marine fishes, the regulatory volume changes are mediated through the actions of ions (Na^+^, K^+^, Cl^−^, Ca^2+^) and water channels (aquaporins) expressed in the spermatozoon plasma membrane [6,7]. Current evidence suggests, however, that the aquaporins involved in RVD and RVI may differ between the lineages. In mammals, AQP3 and AQP8 localized in the spermatozoon tail are considered the main conduits for RVD-mediated water efflux [7,8,9], while AQP3 is also involved in hydrogen peroxide (H_2_O_2_) flux [10,11]. In the post-activated spermatozoa of teleost fishes, however, the AQP8 ortholog Aqp8bb is specifically a mitochondrial peroxiporin mediating H_2_O_2_ efflux to sustain ATP production for motility maintenance [12,13]. Similarly, recent evidence has shown that marine teleost spermatozoon shrinkage is initially mediated via Aqp1aa, while the ensuing RVI requires the cooperative activity of Aqp4a and the volume-sensitive calcium channel Trpv4, in conjunction with other ion channels and transporters [6,14].

In murine sperm, RVD failure is associated with increased tail angulation at the residual cytoplasmic droplet and infertility in naturally mating mice models [15]. This phenotype has been identified in AQP3-deficient sperm, in which motility activation occurs normally, but sperm progression within the female oviduct is hampered, thus resulting in reduced fertility [7,16]. Teleosts harbor two *AQP3* paralogs, *aqp3a* and −*3b*, that arise through whole genome duplication (WGD) events, although additional non-WGD lineage-dependent duplicates are also present in some species [17]. In freshwater teleosts, the spermatozoon mRNA expression of *aqp3a* has only been reported in the cyprinid *Gobiocypris rarus* [18], although the localization of the protein product is currently unknown. In marine fish spermatozoa, such as the gilthead seabream (*Sparus aurata*), *aqp3*-encoding transcripts were initially not detected by RT-PCR [19], but a recent transcriptomic study using RNA-seq revealed the mRNA expression of *aqp3a* in mature sperm [20]. In the present work, we examine the localization of Aqp3a in gilthead seabream spermatozoa using a paralog-specific antibody, and utilize Aqp3a-specific chemical and immunological inhibitors to investigate the potential role of the channel during sperm motility.

## 2. Results and Discussion

### 2.1. Aqp3a Is Accumulated during Seabream Sperm Maturation and Localized at the Posterior Flagellum

**Figure 1:**
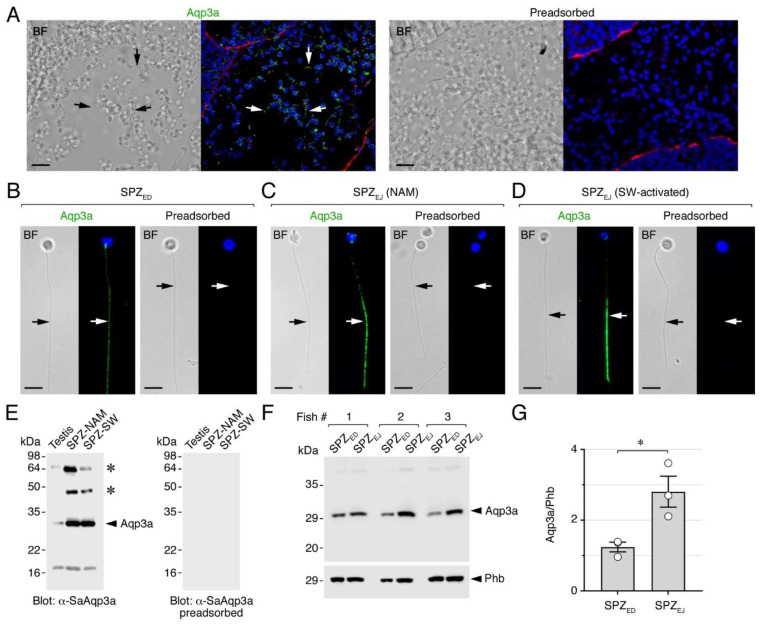
Aqp3a protein expression is upregulated during seabream sperm maturation. (**A**–**D**) Bright field (BF, left) and epifluorescence images (right) of Aqp3a localization in seabream intratesticular sperm (**A**), sperm from the efferent duct (SPZ_ED_), ejaculated sperm (SPZ_EJ_) diluted in non-activating medium (NAM; immotile) and activated sperm in seawater (SW; motile), using a seabream-specific Aqp3a chicken antibody (α-SaAqp3a). In (**A**), sections were counterstained with 4′,6-diamidino-2-phenylindole (DAPI; blue) and fluorophore-coupled lectin wheat germ agglutinin (WGA; red), whereas in (**B**–**D**) the sections were stained with DAPI alone. The right panels in (**A**–**D**) show control sections incubated with the primary antibody preadsorbed by the antigenic peptide to test for specificity. In (**A**), the arrows indicate the spermatozoa, while in (**B**–**D**) the arrows indicate the spermatozoon flagella. Scales bars: 10 µm (**A**), 5 µm (**B**–**D**). (**E**) Immunoblot of protein extracts from immotile and activated sperm probed with α-SaAqp3a. The right panel shows a duplicate blot that was run in parallel and incubated with the preabsorbed antisera. The asterisks indicate potential post-translational modifications of the Aqp3a channel. (**F**) Immunoblot of Aqp3a in SPZ_ED_ and SPZ_EJ_ from three different male fish. Prohibitin (Phb) was used as loading control. In (**E**,**F**), molecular mass markers (kDa) are on the left. (**G**) Densitometric analysis of Aqp3a accumulation in spermatozoa normalized to Phb. Data (mean ± SEM; *n* = 3 males) were statistically analyzed by the unpaired Student’s *t*-test (*, *p* < 0.05). Uncropped blots are shown in Supplementary Figure S1.

As for mammalian AQP3 [21], the orthologous teleost duplicates Aqp3a and -3b are both permeable to water and glycerol [22,23,24,25,26], and can therefore be functionally and phylogenetically classified as typical aquaglyceroporins [17]. In teleosts, Aqp3 channels are expressed in osmoregulatory organs such as the gill, kidney and intestine [27,28], but it is unclear whether these channels are also localized in the spermatozoa as in mammals [11,14]. To clarify whether Aqp3a is present in gilthead seabream sperm, our first step was to determine the localization of this channel during spermatogenesis and isolated sperm cells using immunofluorescence microscopy and a previously validated affinity purified seabream Aqp3a-specific chicken polyclonal antibody (α-SaAqp3a) [26]. In the testis, weak but specific Aqp3a immunostaining was only found in the flagellum of released and differentiated spermatozoa accumulated within the seminiferous tubules, while other germ and somatic cells were negative (Figure 1A). In isolated immature spermatozoa collected from the efferent duct (SPZ_ED_), which arises from the dorsal surface of each elongated testis and connects to the spermatic duct, a weak Aqp3a signal in the posterior half of the tail was noted (Figure 1B). This staining became more intense in ejaculated mature spermatozoa (SPZ_EJ_) diluted in non-activating medium (NAM; immotile) (Figure 1C), and remained unchanged in seawater-activated SPZ_EJ_ (motile) (Figure 1D). The specificity of the reactions in the testicular sections and spermatozoa was confirmed by the preadsorbtion of the α-SaAqp3a with the corresponding immunizing peptide (Figure 1A–D, right panels).

Immunoblot analyses were subsequently carried out to confirm Aqp3a protein expression in both the testis and spermatozoa. The experiments revealed that the α-SaAqp3a detected a reactive band with a molecular mass of ~30 kDa, consistent with the in silico calculated mass of the Aqp3a monomer (33 kDa), in protein extracts from the testis and isolated immotile or activated SPZ_EJ_ (Figure 1E). The intensity of this polypeptide band, which was no longer detected when using the preadsorbed antiserum (Figure 1E, right panel), was much lower in the testis extracts than in the sperm, consistent with the low testicular Aqp3a immunoreaction previously noted using immunofluorescence microscopy. Additional Aqp3a-reactive bands with higher (~45 and ~64 kDa) or lower (~18 kDa) molecular masses were also detected (Figure 1E), which may correspond to posttranslational modifications or degradation products of the channel.

To further investigate whether Aqp3a is accumulated in spermatozoa during their transit through the extratesticular ducts, as suggested from the previous immunostaining experiments, we carried out semiquantitative immunoblot densitometry analyses of Aqp3a. For this, samples of SPZ_ED_ and SPZ_EJ_ were collected from three different males, and the prohibitin (Phb) protein was employed as an endogenous control to normalize the abundance levels of Aqp3a. The data showed that Aqp3a protein expression in SPZ_EJ_ was higher than in SPZ_ED_ (Figure 1F,G), thus confirming that Aqp3a synthesis in spermatozoa is enhanced during their maturation phase in the extratesticular ducts.

Altogether, the above data indicate that seabream Aqp3a is expressed in spermatozoa in a strikingly similar pattern to that reported for mouse and human sperm. In seabream, this channel is localized at the mid posterior region of the spermatozoon flagella, whereas in mice and human AQP3 is mainly confined to the principal piece of the sperm tail [7], although it has also been found along the tail and the base of the head [10,29]. Therefore, in addition to the aquaglyceroporins Aqp7 and -10bb, which are restricted to the head and the anterior tail of the seabream spermatozoon [19], Aqp3a is segregated to the posterior half of the tail, thus reflecting the expression patterns in human sperm where the aquaglyceroporins AQP3 and -7 are compartmentalized in the tail and the base of the head, and the head and midpiece, respectively [29]. As suggested for human sperm [29], these findings may indicate that aquaglyceroporin-mediated permeability could play complementary functions in seabream sperm. The importance of Aqp3a in seabream spermatozoa may also be reinforced by our present finding that *aqp3a* translation is upregulated during sperm maturation. This observation is consistent with the accumulation of many transcripts encoding translational regulators and cytoplasmic and mitochondrial ribosomal proteins during the differentiation and maturation of seabream sperm [20]. In addition, studies in mammals indicate that sperm can perform de novo protein synthesis during the acquisition of hypermotility (capacitation) in the female reproductive tract [30,31,32,33,34]. The activation of Aqp3a translation in fish spermatozoa specifically during the maturation phase should therefore be investigated in more detail in the future.

### 2.2. Specific Chemical and Immunological Inhibition of Seabream Aqp3a Channel Function 

**Figure 2:**
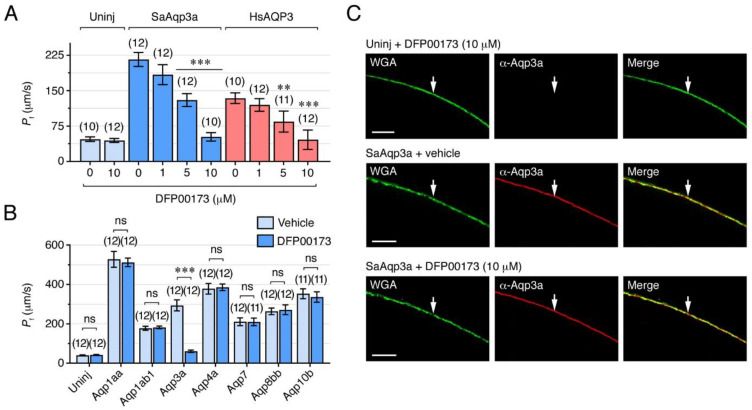
The DFP00173 specifically inhibits seabream Aqp3a (SaAqp3a) water conductance in *X. laevis* oocytes. (**A**) Inhibition of *P*_f_ of *X. laevis* oocytes noninjected (Uninj, light blue) or expressing SaAqp3a (blue) and exposed to 0.5% DMSO alone or containing increasing concentrations of DFP00173. Oocytes expressing human (HsAQP3) were used as positive controls (red). (**B**) Effect of DFP00173 (10 µM) or vehicle (0.5% DMSO) on the *P*_f_ of noninjected oocytes or expressing different seabream aquaporins that are expressed in the seabream spermatozoa. In (**B**,**C**) data are the mean ± SEM (*n* indicated above each bar) and were statistically analyzed by one-way ANOVA or an unpaired Student’s *t*-test, respectively (**, *p* < 0.01; ***, *p* < 0.001; with respect to oocytes not exposed to the inhibitor, or as indicated in brackets). ns, statistically not significant. (**C**) Immunolocalization of SaAqp3a in noninjected oocytes and oocytes expressing SaAqp3a and treated or not with DFP00173 (10 µM). The plasma membrane, indicated by an arrow, was stained with wheat germ agglutinin (WGA; green). Scale bars, 10 µm.

To investigate the role of Aqp3a during sperm motility, we first evaluated the efficiency of different pharmacological and immunological approaches to specifically block the activity of the channel. For the pharmacological inhibition of Aqp3a, we tested the efficiency and selectivity of the ethyl 4-(carbamoylamino)benzoate compound DFP00173, which has recently been shown to selectively inhibit water, glycerol and peroxide permeability of mouse and human AQP3 [35]. To do this, we heterologously expressed Aqp3a in *Xenopus laevis* oocytes and determined aquaporin-mediated water permeability in the presence or absence of DFP00173. Accordingly, oocytes were injected with Aqp3a or human AQP3 cRNAs, or not injected (negative controls), and exposed to increasing amounts of DFP00173 (1, 5 or 10 µM) or 0.5% DMSO (controls) for 30 min, and their water permeability subsequently assessed after direct exposure to a hyposmotic medium in the presence of the inhibitor. Application of DFP00173 resulted in a reduction of the osmotic water permeability (*P*_f_) of Aqp3a- and AQP3-expressing oocytes in a dose-dependent manner, whereas that of the negative controls treated with the highest DFP00173 dose was unaffected (Figure 2A). The DFP00173 inhibited the *P*_f_ of the Aqp3a oocytes by 40 ± 6% (mean ± standard error of the mean [SEM]) and 76 ± 4% at 5 and 10 µM, respectively, while the inhibitor reduced the water permeability of the AQP3 oocytes by 37 ± 5% and 66 ± 4% at the same doses.

To assess whether the DFP00173 inhibitor was selective towards Aqp3a, uninjected oocytes and oocytes expressing Aqp3a or each of the Aqp1aa, -1ab1, -4a, -7, -8bb or -10bb paralogs also present in seabream sperm [6,19], were exposed to 10 µM DFP00173 or 0.5% DMSO (control vehicle) and their water permeability determined. The DFP00173 exclusively reduced the *P*_f_ of oocytes expressing Aqp3a (Figure 2B), thus showing that the inhibitor selectively and efficiently blocks the Aqp3a paralog within the group of seabream aquaporins present in spermatozoa. Subsequent immunostaining of Aqp3a-expressing oocytes with the α-SaAqp3a, together with wheat germ agglutinin (WGA) labeling to stain the oocyte plasma membrane, revealed that the channel was completely targeted to the plasma membrane, whereas no staining was detected in the uninjected control oocytes (Figure 2C). The data also showed that the Aqp3a immunoreaction in the membrane was independent of DFP00173 treatment, suggesting that Aqp3a remains in the plasma membrane during the exposure to the inhibitor. The DFP00173 therefore appears to specifically inhibit Aqp3a-dependent water permeability in a manner distinct from internalization, possibly by blocking the channel pore, as has been described for the mammalian AQP3 [35].

**Figure 3:**
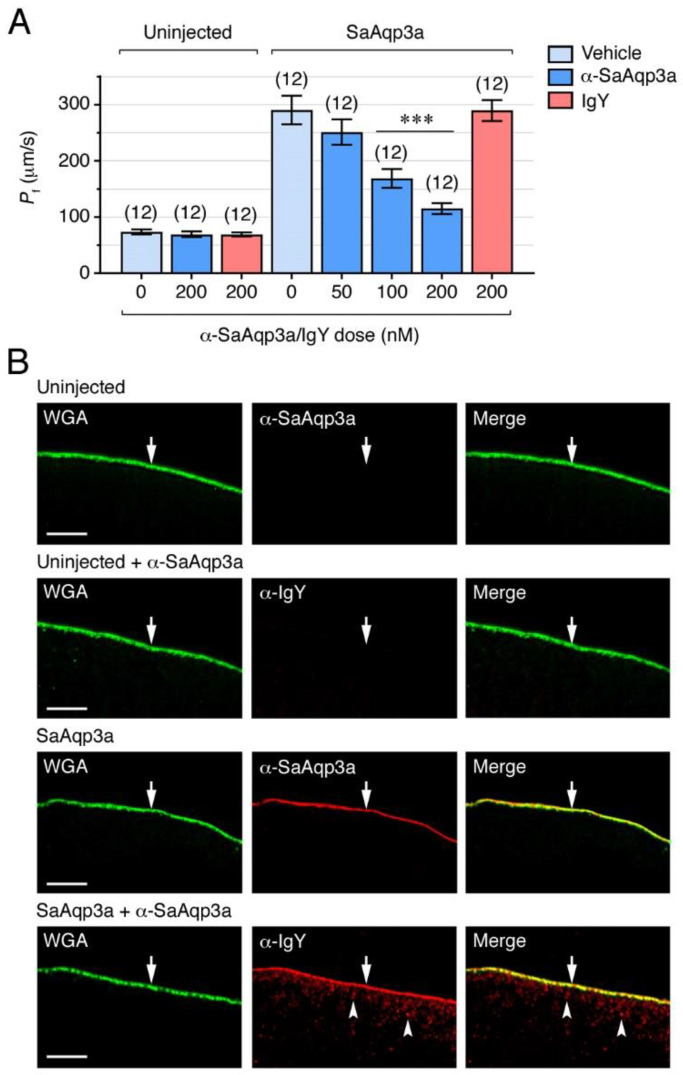
Immunological inhibition of seabream Aqp3a in *X. laevis* oocytes. (**A**) Water permeabilities of uninjected oocytes or oocytes expressing SaAqp3a in the presence of 0.5% DMSO alone (light blue) or with increasing amounts of the seabream Aqp3a-specific antibody (α-SaAqp3a, blue). Immunoglobulin Y (IgY) was used as the negative control (red). The data are presented as the mean ± SEM (*n* indicated above each bar) and were statistically analyzed by one-way ANOVA (***, *p* < 0.001, with respect to non-treated Aqp3a-expressing oocytes). (**B**) Immunolocalization of SaAqp3a in noninjected oocytes or expressing SaAqp3a and treated or not with 200 nM of α-SaAqp3a. Sections from Uninj oocytes and oocytes expressing SaAqp3a and not treated with the α-SaAqp3a, were probed with the primary (α-SaAqp3a) and secondary (α-IgY) antibodies, whereas oocytes treated with the α-SaAqp3a antibody in vitro were probed only with the secondary α-IgY. The plasma membrane (arrows) was stained with wheat germ agglutinin (WGA; green). The arrowheads indicate partial cytoplasmic retention of Aqp3a in the presence of the antibody. Scale bars, 10 µm.

For the immunological inhibition of Aqp3a function, we tested the suitability of the α-SaAqp3a to inhibit channel-mediated water transport. For this, uninjected oocytes and oocytes expressing Aqp3a were exposed to 0.5% DMSO, increasing external concentrations of α-SaAqp3a (50, 100 and 200 nM) or 200 nM of chicken immunoglobulin Y (IgY; control antibody) for 1 h prior to *P*_f_ measurements under hypotonic conditions as described above. The data showed that the water permeability of Aqp3a-expressing oocytes was inhibited by α-SaAqp3a in a dose-response manner up to 60 ± 3% (with 200 nM), but unaffected by treatment with IgY (Figure 3A). Staining of Aqp3a-expressing oocytes previously exposed to α-SaAqp3a with only Cy3-labelled anti-chicken IgY secondary antibodies and WGA revealed that in these oocytes Aqp3a remained in the plasma membrane but was partially retained in the cortical cytoplasm (Figure 3B). These data indicate that, in contrast to the DFP00173 inhibitor, the α-SaAqp3a partially prevented the trafficking of the channel to the oocyte surface. Our observations thus suggest that exogenous addition of α-SaAqp3a can specifically bind Aqp3a and inhibit its function, possibly through steric disruption of the trafficking mechanism. However, since in α-SaAqp3a-treated oocytes the anti-chicken IgY also stained the plasma membrane, it is also plausible that binding of the antibody to the Aqp3a C-terminus may reduce the permeability of plasma membrane-inserted Aqp3a by disrupting the channel pore from the intracellular side. In any event, the specific mechanism by which the α-SaAqp3a inhibits Aqp3a function is yet unclear and should be investigated in the future.

### 2.3. Aqp3a Inhibition Impairs Seabream Sperm Motility

**Figure 4:**
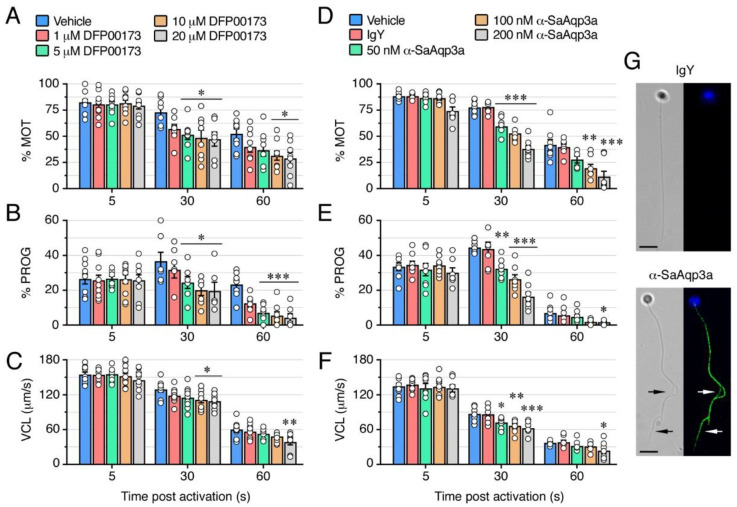
Inhibition of Aqp3a impairs seabream sperm motility. (**A**–**F**) Dose-response inhibition of the percentage of motility and progressivity (% MOT and % PROG, respectively) and curvilinear velocity (VCL) at 5, 30 and 60 s post activation induced by DFP00173 (**A**–**C**) and the seabream Aqp3a antibody (α-SaAqp3a) (**D**–**F**). Control spermatozoa were treated with 0.5% DMSO (vehicle) or 200 nM IgY. In all panels, the data (*n* = white dots above each bar, corresponding to one ejaculated per male) are presented as the mean ± SEM. Statistical differences within each time point were measured by one-way ANOVA, or Kruskal-Wallis test, followed by Dunn’s multiple comparisons test (*, *p* < 0.05; **, *p* < 0.01; ***, *p* < 0.001, with respect to DMSO-treated sperm). (**G**) Sperm treated with IgY or α-SaAqp3a, further activated for ~30 s, and subsequently stained only with anti-chicken secondary antibodies, confirmed the specific binding of α-SaAqp3a to its target protein (green) in the spermatozoon flagellum (arrows). The nuclei of the spermatozoa were counterstained with DAPI (blue). Scale bars, 5 µm.

By using the DFP00173 inhibitor and α-SaAqp3a, we subsequently investigated the functional role of Aqp3a during seabream sperm motility. In the first experiments, immotile SPZ_EJ_ were preincubated with increasing concentrations of DFP00173 (1, 5, 10 or 20 µM) or 0.5% DMSO vehicle (controls), for 30 min, and motility was further activated in seawater containing the same amounts of inhibitor and vehicle. The effect of the treatments on sperm motility was determined by analyzing different kinetic parameters, percentage of motility and progressive spermatozoa (% MOT and % PROG, respectively), and the curvilinear velocity (VCL), at 5, 30 and 60 s post activation using computer-assisted sperm analysis (CASA). None of the DFP00173 concentrations tested reduced the % MOT, % PROG and VCL at 5 s post activation (Figure 4A–C). However, at 30 s, a clear inhibitory effect on the motility and progressivity of spermatozoa was observed with 5, 10 and 20 µM of DFP00173, which reached 36 ± 9% and 59 ± 9%, respectively, with the highest dose (Figure 4A,B). In contrast, the VCL was reduced only with doses ≥ 10 µM, being inhibited by 16 ± 3% with 20 µM of DFP00173 (Figure 4C). At 60 s post activation, the inhibitory effect of DFP00173 on the kinetics of spermatozoa was more efficient, since 20 µM of the inhibitor reduced the % MOT, % PROG and VCL by 58 ± 10%, 82 ± 9% and 35 ± 8%, respectively (Figure 4A–C).

To test the effect of Aqp3a immunological inhibition on sperm motility, SPZ_EJ_ were preincubated in NAM containing increasing doses of α-SaAqp3a (50, 100 or 200 nM), 200 nM IgY (control antibody), or 0.5% DMSO (control vehicle) for 1 h prior to activation, and the sperm kinetics were determined as above in the absence of the antibody. As observed for DFP00173, the α-SaAqp3a did not reduce the sperm motility at 5 s post activation, while inhibitory effects of the antibody were seen at 30 and 60 s post activation (Figure 4D–F). At 30 s, the α-SaAqp3a induced a dose-response inhibition of the motility parameters of spermatozoa with respect to those exposed to DMSO alone, whereas treatment with IgY had no effect. Thus, the antibody reduced the % MOT, % PROG and VCL by, respectively, 23 ± 4% and 51 ± 4% (from 50 to 200 nM, respectively), 27 ± 4% and 63 ± 6% (from 50 to 200 nM, respectively), and 17 ± 4% and 28 ± 4% (from 50 to 200 nM, respectively) (Figure 4D–F). At 60 s post-activation time, the control spermatozoa motility had declined compared to the previous experiments, and consequently the % MOT was only reduced with the highest doses of α-SaAqp3a (53 ± 9% and 73 ± 12% inhibition with 100 and 200 nM, respectively), whereas the % PROG and VCL were only blocked with 200 nM (79 ± 5% and 36 ± 13% inhibition, respectively) (Figure 4D–F).

To further confirm the specificity of the immunological inhibition on sperm motility, SPZ_EJ_ treated with 200 nM IgY or α-SaAqp3a and subsequently activated for 30 s were fixed and labeled only with TRITC-coupled anti-chicken IgY secondary antibodies. Fluorescence signals in spermatozoa treated with α-SaAqp3a were observed in the deformed mid-posterior region of the spermatozoon flagella, whereas the sperm treated with IgY were negative (Figure 4G). This staining pattern mimicked the distribution of Aqp3a previously found on fixed SPZ_EJ_, and therefore suggests that the α-SaAqp3a specifically bound its target protein in vitro.

The present results employing two independent and specific methods to block Aqp3a function in seabream spermatozoa therefore suggest that this channel is not required for motility activation, but is necessary for the post-activated maintenance of swimming performance, as also observed for Aqp1ab1, -4a, -7 and -8bb [6,12,13,36].

### 2.4. Aqp3a Dysfunction Induces Tail Deformations in Post-Activated Spermatozoa

**Figure 5:**
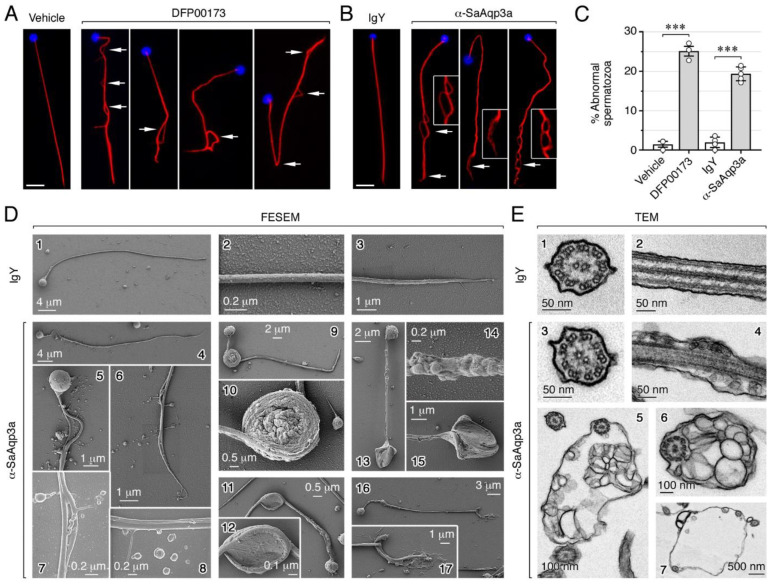
Chemical and immunological inhibition of Aqp3a induces abnormal sperm tail morphology. (**A**–**B**) Representative immunostaining of α-tubulin in seawater-activated spermatozoa treated with vehicle (0.5% DMSO), 20 µM DFP00173, or 200 nM IgY or α-SaAqp3a at ~30 s post activation time, showing that both Aqp3a inhibitors generate sperm tail bending and local swelling in the spermatozoon. Scale bars, 5 µm. (**C**) Percentage of spermatozoa showing sperm tail structural defects. Data (*n* = white dots above each bar, corresponding to one ejaculated per male) are presented as the mean ± SEM, and were statistically analyzed by an unpaired Student *t*-test (***, *p* < 0.001, with respect to vehicle- or IgY-treated sperm). (**D**,**E**) Field emission scanning electron microscope (FESEM; (**D1–17**)) and transmission electronic microscopy (TEM, (**E1–7**)) revealed that activated sperm treated with α-SaAqp3a show expanded intracellular space in the flagella compared with the IgY-exposed sperm, as well as a progressive volume expansion and tail bending. In some cases, the sperm tail also becomes strongly coiled. Scale bars are indicated in each panel.

The previous fluorescent staining experiments revealed that the α-SaAqp3a, but not IgY, induced deformities in the spermatozoon tail (Figure 4G, lower panel), which we did not observe in previous studies using antibodies against Aqp1aa, -1ab1, -4a, -7 or -8bb on seabream sperm motility assays [6,12,36]. To confirm this, SPZ_EJ_ treated with 20 µM DFP00173 or 200 nM α-SaAqp3a, as well as the corresponding controls exposed to vehicle and IgY, respectively, were immunostained with an anti-tubulin monoclonal antibody at ~30 s post activation time. Control spermatozoa exhibited a normal morphology, with a rounded head and a straight flagellum, whereas ~20–25% of spermatozoa exposed to DFP00173 and α-SaAqp3a showed bending and local swelling of the spermatozoon tail, mostly towards the mid-posterior region of the flagella where Aqp3a is mainly localized (Figure 5A–C). These morphological abnormalities of the sperm flagella therefore appear to be specifically induced when Aqp3a function is impaired and are linked to a progressive post-activation asthenoteratozoospermia.

To examine the ultrastructural flagellar defects resulting from Aqp3a inhibition, which could shed light on the underlying mechanisms, we carried out field emission scanning electron microscopy (FESEM) and transmission electron microscopy (TEM) of post-activated spermatozoa exposed to IgY or α-SaAqp3a as above (Figure 5). Due to the rapid effects of the inhibitor on the sperm morphology, which can be easily altered or damaged by conventional electron microscopy methods, we employed protocols for rapid sample preparation and adequate fixative solutions to maintain the original ultrastructure details while avoiding the presence of artifacts. In addition, the external morphology of the spermatozoa was evaluated without a coating layer to ensure a nearly native cell surface structure, with FESEM operating at low voltage and high-speed scanning conditions to reduce beam damage on the samples. Under these conditions, scanning and transmission electron microscopy image analyses revealed that in comparison to the IgY-exposed sperm (Figure 5(D1–D3,E1,E2)), spermatozoa treated with α-SaAqp3a showed an increased cell swelling in the flagella (Figure 5(D4–D8,D13,D14,E3,E4)). In some cases, there was clear angulation of the sperm tail and the formation of a hairpin-like structure (Figure 5(D9–D12)). In several swelling sperm cells, the hairpin portion of the bending sperm tail, which also occurred at the end of the flagellum, was encapsulated within the stretched membrane, creating an increased intracellular volume, while in others the flagella became strongly coiled (Figure 5(D9–D12,D15,D16,E5–E7)). These observations suggest that seabream sperm with disrupted Aqp3a function failed to regulate cell volume under the high hyperosmotic stress imposed by seawater, which was likely responsible for the observed tail deformations and the subsequent reduced motility of post-activated sperm.

### 2.5. A Role of Aqp3a during Seabream Sperm Osmoadaptation

The data from the present study suggest that Aqp3a is an essential membrane pathway for the osmoadaptation of seabream sperm. However, the exact mechanisms by which Aqp3a regulates cell volume, allowing the RVI response after seawater activation of motility, are not yet known. The observed tail abnormalities observed in seabream spermatozoa with pharmacologically and immunologically inhibited Aqp3a are strikingly similar to those reported in *AQP3*-deficient murine sperm, which show impaired RVD in response to physiological hypotonic stress in the female oviduct [7]. Imaging of both ultrastructural studies and time-lapse revealed that in murine sperm, increased cell swelling begins at the residual cytoplasmic droplet, forcing the tail to angulate and form a hairpin-like structure due to mechanical membrane stretch, thus reducing sperm motility [7]. Currently, the molecular mechanisms by which AQP3 may regulate RVD in mammalian sperm are not known. It has been speculated that this channel could act as a specialized osmo-/mechanosensing system in conjunction with yet unknown volume-sensitive ion channels for the initial sensing of cell swelling and the triggering of water efflux for the RVD response [7,16]. In seabream spermatozoa, both Aqp4a and Trpv4 cooperatively regulate an RVI response under high hypertonic stress, which also requires the interplay with other ion channels and transporters [6]. Therefore, a similar mechanism could be hypothesized for Aqp3a through its interaction with other ion channels to sense mechanical cues in post-activated spermatozoa, thereby controlling water fluxes for cell volume regulation and motility maintenance.

However, an alternative hypothesis to explain the abnormal swelling of the seabream sperm tail could be the accumulation of osmolytes, such as glycerol, which cannot be released across the plasma membrane when Aqp3a function is inhibited. In human sperm, DFP00173-mediated blockage of AQP3 has not been associated with a higher occurrence of tail defects [29]. Rather, it has been proposed that this channel may be specialized in H_2_O_2_ flux and the triggering of various signaling pathways during capacitation [10,11]. On the contrary, AQP7 appears to be the main aquaglyceroporin responsible for the facilitated transport of glycerol into human sperm cells, which may be an important substrate for sperm bioenergetics during capacitation, although the involvement of AQP7 in the modulation of yet unknown transduction pathways or mechanisms may also be possible [29]. In fish, the activation of sperm hypermotility, a mechanism that is reminiscent of the capacitation process of mammalian spermatozoa [37], occurs upon release into seawater, and therefore, Aqp3a-mediated glycerol movement into activated sperm cells seems not to be a plausible mechanism. However, studies in teleosts have shown that the energy-supplying pathways in SPZ_EJ_, in addition to glycolysis and oxidative phosphorylation, also include phospholipid catabolism and triglyceride metabolism [38,39], which may lead to the formation of high intracellular levels of glycerol. Accordingly, in seabream SPZ_EJ_, the mRNA expression of different enzymes involved in triglyceride and lipid metabolism is upregulated [6]. It can therefore be hypothesized that an Aqp3a-mediated efflux of intracellular glycerol that is accumulated in association with ATP storage in maturing spermatozoa during their transit through the extratesticular ducts is required in post-activated spermatozoa. Indeed, intracellular osmolyte concentrations initially increase due to the rapid Aqp1aa-mediated water efflux and cell shrinkage required for sperm motility activation [6,36]. Consequently, the inhibition of selective osmolyte efflux via Aqp3a during the ensuing RVI phase could result in hyperhydration through Aqp1aa or -4a, both distributed along the tail, leading to an aberrant swelling of the flagellum, thus compromising the motility of post-activated spermatozoa.

## 3. Materials and Methods

### 3.1. Animals and Sample Collection

Adult fish 2–3 years old were housed at the Institut de Ciències del Mar (CSIC, Barcelona, Spain) according to established protocols [19]. Samples of SPZ_EJ_ were collected during the natural reproductive season (November–February) through gentle abdominal pressure on sedated (500 ppm of 2-phenoxyethanol) male fish using a syringe in the gonopore to prevent seawater or urine contamination. Subsequently, fish were euthanized by an overdose of the anesthetic followed by decapitation to recover samples of SPZ_ED_ and biopsies of the testis. Adult *X. laevis* were purchased from the Centre de Ressources Biologiques Xénopes (University of Rennes, France) and maintained at the AQUAB facilities of the Universitat Autònoma de Barcelona (UAB, Spain). Frogs were maintained in filtrated freshwater at 18°C, under a 12-h light-dark cycle, and fed two days a week with beef heart or pellets (*Xenopus* Sticks, AQUA Schwarz GmbH, Göttingen, Germany). Oocytes were collected via surgical laparotomy from anesthetized females. Procedures relating to the care and use of fish and sample collection, and for surgical laparotomy of female frogs, were approved by the Ethics Committee of CSIC, the Ethics Committee for Animal and Human Experimentation from UAB, and the Catalan Government (Direcció General de Polítiques Ambientals i Medi Natural; Projects no. 10985 and 12147).

### 3.2. Chemicals and Antibodies 

The AQP3-specific inhibitor DFP00173 was purchased through MedChemExpress (Monmouth Junction, NJ, USA). All other chemicals were purchased from Merck KGaA (Darmstadt, Germany), unless stated otherwise. The affinity-purified chicken polyclonal antibody specific for seabream Aqp3a has been characterized elsewhere [26]. The Phb antibody was purchased from GeneTex (# GTX124491; Irvine, CA, USA), whereas the anti-α-tubulin mouse monoclonal antibody was from Merck (T9026). The secondary antibodies goat anti-chicken IgY coupled with Alexa Fluor 488, horseradish peroxidase-coupled anti-chicken IgY, and Alexa Fluor 555-coupled goat anti-mouse were from Invitrogen (# A-11039, # PA1-28798 and # A-21422 (Carlsbad, CA, USA), respectively; Waltham, MA, USA).

### 3.3. Oocyte Swelling Assays

The different aquaporin cDNAs were heterologously expressed in *X. laevis* oocytes as previously described [40]. Oocytes were injected with 10 ng of cRNA, whereas the control oocytes were not injected. The changes in oocyte *P*_f_ were recorded in ten times diluted Modified Barth’s Solution (MBS: 88mM NaCl, 1 mM KCl, 2.4 mM, NaHCO_3_, 0.82 mM MgSO_4_, 0.33 mM Ca(NO_3_)_2_, 0.41 mM CaCl_2_, 10 mM HEPES and 25 μg/mL gentamycin) at pH 7.5, or at pH 8.5 for Aqp3a-expressing oocytes. To test the effect of DFP00173, oocytes were preincubated in MBS containing increasing amounts of the compound (0, 1, 5, or 10 µM) and 1% DMSO for 30 min at room temperature prior to the swelling assays, which were carried out under the same concentrations of DMSO and DFP00173. Control oocytes were exposed to 1% DMSO alone. For the immunological inhibition of Aqp3a-mediated water transport, uninjected and injected oocytes were preincubated with increasing concentrations of α-SaAqp3a (50, 100, or 200 nM), or 200 nM of chicken IgY, in the presence of 0.5% DMSO, for 1 h at 18°C prior to the swelling assays. Control oocytes were treated with 0.5% DMSO alone. 

### 3.4. Sperm Motility Measurements

Freshly collected sperm was 1:100 diluted in NAM (in mg/mL: 3.5 NaCl, 0.11 KCl, 1.23 MgCl_2_, 0.39 CaCl_2_, 1.68 NaHCO_3_, 0.08 glucose, 1 bovine serum albumine, pH 7.7; 280 mOsm) supplemented with 200 μg/mL gentamycin (Invitrogen). The sperm concentration and kinematic properties were determined using the Integrated Semen Analysis System (ISAS^®^v1, Proiser, Valencia, Spain) software, as described previously [19]. Activation of sperm (10^8^ cells/mL) was carried out at 1:10 dilution in filtered seawater, and motility parameters were recorded at 5, 30 and 60 s post activation. The effect of DFP00173 on sperm motility was determined by activating sperm with seawater in the presence or absence of increasing concentrations (1, 5, 10 or 50 µM) of the inhibitor. To investigate the impact of α-Aqp3a on sperm motility, immotile sperm diluted in NAM were incubated with increasing doses of the antibody (50, 100 or 200 nM), or 200 nM of chicken IgY (control), in the presence of 0.5% DMSO for 1 h at 16 °C prior to activation in seawater. In all experiments, control sperm were treated with 0.5% DMSO, a concentration previously observed not affecting the motility of seabream spermatozoa [12,36]. Sperm motility parameters and CASA settings were as reported previously [41]. Only fresh sperm exhibiting > 80% motility were selected for the experiments, and the sperm kinematic parameters were recorded in triplicate for each ejaculate.

### 3.5. Protein Extraction and Immunoblotting

For protein extracts from the whole spermatozoa, freshly collected samples from the efferent duct or from the ejaculate were diluted in NAM (10^9^ cells/mL) and mixed with ice-cold 2 × RIPA buffer (300 mM NaCl, 100 mM Tris-HCl pH 8, 2% Triton X-100, 1% sodium deoxycholate, 2 mM EDTA, 2 mM EGTA, 2 mM Na_3_VO_4_, 2 mM NaF, 200 U of benzonase, and EDTA-free protease inhibitors). Activated spermatozoa (10^9^ cells/mL) were collected after exposure of NAM-incubated sperm to seawater as described above. The cells were dissociated using a glass dounce homogenizer, followed by centrifugation at 14,000× *g* for 10 min at 4 °C. The resulting supernatant was combined with 2 × Laemmli sample buffer (containing 5% β-mercaptoethanol), heated at 95 °C for 15 min, frozen in liquid nitrogen, and stored at −80 °C. 

For immunoblotting, protein extracts were denatured at 95 °C for 10 min, electrophoresed in 12% SDS-PAGE, and transferred onto Immun-Blot nitrocellulose 0.2 μm membranes (Bio-Rad Laboratories, Inc., Hercules, CA, USA) as described previously [19]. Membranes were blocked with 5% non-fat dry milk or 3% bovine serum albumin (BSA) in TBST (20 mM Tris, 140 mM NaCl, 0.1% Tween, pH 7.6). Incubation with the α-Aqp3a (1:500 dilution) was performed overnight at 4ºC. Bound antibodies were detected using horseradish peroxidase-coupled anti-chicken IgY antibodies for 1 h at room temperature. Following TBST washing, immunoreactive bands were revealed using the C-DiGit Blot Scanner (LI-COR Biosciences-GmbH, Madrid, Spain) and WesternSure PREMIUM (# 926-95000; LI-COR Biosciences-GmbH, Spain) as the chemiluminescent substrate. To verify anti-Aqp3a antibody specificity in spermatozoa, the antibody was preabsorbed with its corresponding peptide (at a 1:10 ratio) for 1 h at 37 °C before membrane incubation. Quantification of protein expression was made using Phb as the reference protein and the software (Image Studio 5.0) provided by the manufacturer of the blot scanner.

### 3.6. Immunofluorescence Microscopy

Testis biopsies and *X. laevis* oocytes were fixed for 6 h in 4% paraformaldehyde (PFA) in phosphate buffer saline (PBS; 137 mM NaCl, 2.7 mM KCl, 100 mM Na_2_HPO_4_, 2 mM KH_2_PO_4_, pH 7.4) and subsequently dehydrated and mounted in paraffin as described previously [42]. The sections (8-µm thick) were rehydrated and blocked with PBS containing 5% normal goat serum and 0.1% BSA for 1 h at room temperature and incubated with the Aqp3a antiserum (1:400 in PBS) overnight at 4 °C in a humidified chamber. Following three washes with PBS, the slides were incubated with goat anti-chicken IgY coupled with Alexa Fluor 488 (1:800 diluted in PBS) for 1 h at room temperature. After another wash with PBS, the sections were counterstained with WGA Alexa Fluor^®^ 647-conjugated (# W32466; Life Technologies Corp., Carlsbad, CA, USA) for 10 min (dilution 1:10,000 in PBS) and 4′,6-diamidino-2-phenylindole (DAPI, # G8294, Merck; dilution 1:5000 in PBS) for 3 min. The sections were then mounted with fluoromount aqueous anti-fading medium (# F4680, Merck), and images were captured using a Zeiss Axio Imager Z1/ApoTome fluorescence microscope (CarlZeiss Corp., Oberkochen, Germany).

For spermatozoa, cells were attached to UltraStick/UltraFrost Adhesion slides (Electron Microscopy Sciences, Hatfield, PA, USA) and activated with seawater before being fixed directly on the slide in 4% PFA in PBS for 15 min. The slides were rinsed in PBS and subsequently the cells permeabilized using boiling citrate at 0.01 M and pH 6 for 5 min, repeated three times. After cooling the citrate solution to room temperature, the slides were washed in PBS and incubated with 0.2% Triton X-100 for 15 min at room temperature. After blocking with PBS containing 5% normal goat serum and 0.1% BSA for 1 h at room temperature, slides were incubated with the primary and secondary antibodies as described above. For the immunostaining of tubulin, the primary and secondary antibodies were used at 1:1000 and 1:2000 dilutions, respectively. In all cases, the nuclei were subsequently counterstained with DAPI for 3 min. Finally, the slides were mounted and photographed following the procedure indicated above.

### 3.7. Electron Microscopy

For the imaging of external morphology and cell surface with FESEM, sperm cells of IgY and α-App3a were deposited on cover glasses, fixed with 2.5% glutaraldehyde in 0.1 M phosphate buffer (PB) during 1 h, rinsed in PB, post-fixed in 1% osmium tetroxide 0.8% potassium ferrocyanide in PB, rinsed in deionized water, dehydrated in graded series of ethanol, and dried in a critical-point dryer (Bal-Tec AG, Balzers, Liechtenstein) with CO_2_. Samples were mounted in metallic stubs and observed without coating in a FESEM Zeiss Merlin (Carl Zeiss, Oberkochen, Germany) operating at 1 kV and equipped with a secondary electron (SE) detector. Representative micrographs of entire sperm cells and tails were randomly acquired at different magnifications (from 5000× to 100,000×). 

For the imaging of internal ultrastructure with TEM, the same samples were pelletized, fixed with 2.5% glutaraldehyde and 2% paraformaldehyde in PB, rinsed in PB, post-fixed in 1% osmium tetroxide/0.8% potassium ferrocyanide in PB, rinsed in deionized water, dehydrated in graded series of acetone, embedded in Epon resin (Ted Pella Inc., Redding, CA, USA) and polymerized for 48 h at 60 °C. Ultrathin sections (70 nm) of selected areas were placed on 200-mesh copper grids, contrasted with uranyl acetate and lead citrate, and observed in a TEM Jeol JEM-1400 (Jeol Ltd., Tokyo, Japan) operating at 80 kV and equipped with an Orius SC 200 CCD camera (Gatan, Abingdon, UK). Representative micrographs of tails were randomly acquired at different magnifications (from 5000× to 200,000×).

### 3.8. Statistical Analysis

Data are expressed as mean ± SEM, and percentages were square root transformed prior to analyses. Data were tested for both normal distribution and homogeneity of variance by the Kolmogorov-Smirnov and Bartlett’s tests, respectively. Statistical differences among multiple groups were analyzed by one-way ANOVA, or the nonparametric Kruskal-Wallis test, followed by the Dunnett’s multiple comparison test or the Dunn’s test, respectively. Pairwise comparisons were made by the two-tailed unpaired Student’s *t*-test or by the nonparametric Mann Whitney’s *U*-test. Statistical analyses were performed using GraphPad Prism v9.4.1 (681) software (GraphPad Software). In all cases, statistical significance was defined as *p* < 0.05 (*), *p* < 0.01 (**), or *p* < 0.001 (***).

## 4. Conclusions

The present work identifies Aqp3a, an aquaglyceroporin membrane channel, as a potential osmo-/mechanosensor located at the mid posterior region of the flagella of marine teleost spermatozoa that regulates cell volume. Chemical and immunological inhibition of the channel do not affect activation of motility but do reduce the swimming kinematics of post-activated spermatozoa. The expression pattern of Aqp3a and the phenotypic basis underlying the asthenoteratozoospermia resulting from its inhibition are highly reminiscent of AQP3-defficient murine sperm, whereby local swelling and tail angulation arise in the flagellum. These observations suggest that the respective roles of Aqp3a and AQP3 in marine teleost and murine sperm osmoadaptation may be conserved, despite that they are activated under different osmotic conditions.

## Data Availability

All related data are included in either the manuscript or Supplementary Information. Other data are available from the corresponding author upon request.

## Supplementary Materials

The following supporting information can be downloaded at: https://www.mdpi.com/article/10.3390/ijms25179604/s1.
