# Supplementary figures and images for: Aquaporin-3a Dysfunction Impairs Osmoadaptation in Post-Activated Marine Fish Spermatozoa
Source: Int J Mol Sci. 2024 Sep 4;25(17):9604. doi: 10.3390/ijms25179604 (PMC11395232; doi:10.3390/ijms25179604)

Figure 1E

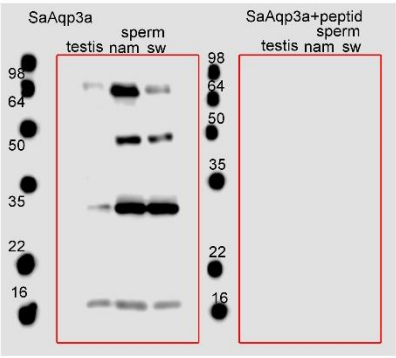

Figure 1F

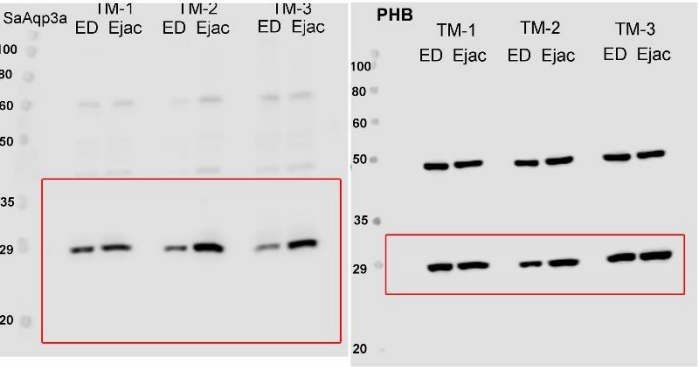

Figure S1. Uncropped blots shown in Figure 1.

Supplement: Supplementary file 1 [file ijms-25-09604-s001.zip › ijms-3173554-supplementary.pdf]
